# The Systemic Inflammation Index: A New Candidate Minor Criterion in the Diagnosis of Polycythemia Vera

**DOI:** 10.3390/jpm14050471

**Published:** 2024-04-29

**Authors:** Emine Gulturk, Korhan Kapucu

**Affiliations:** Department of Hematology, Bakirkoy Sadi Konuk Training and Research Hospital, 34147 Istanbul, Turkey; korhan_kapucu@hotmail.com

**Keywords:** polycythemia vera, secondary polycythemia, systemic inflammation index, erythropoietin, neutrophil-to-lymphocyte ratio, platelet-to-lymphocyte ratio

## Abstract

Aim: To investigate inflammation indices and erythropoietin levels for their potential role in distinguishing polycythemia vera from secondary polycythemia and to compare different parameter combinations in terms of the diagnostic accuracy. Methods: This retrospective cohort was created from patients assessed for polycythemia from January 2020 to December 2023. Polycythemia vera diagnosis was made according to the 2016 World Health Organization criteria (*n* = 145). Those who did not fulfill the criteria were defined as having secondary polycythemia (*n* = 84). Results: The neutrophil lymphocyte ratio, platelet lymphocyte ratio and systemic immune-inflammation index were significantly higher in the polycythemia vera group (*p* < 0.001 for all). Erythropoietin had the highest area under the curve in the analysis to distinguish groups, followed by the systemic immune-inflammation index. The platelet lymphocyte ratio (≥135) had the highest specificity to detect polycythemia vera, followed closely by the systemic immune-inflammation index. The sensitivity for polycythemia vera detection was highest with the erythropoietin and systemic immune-inflammation index combination, followed by erythropoietin and the neutrophil lymphocyte ratio. All the single and combinatory variables exhibited significant performance in predicting polycythemia vera after adjusting for age and sex. However, the erythropoietin and systemic immune-inflammation index combination had the highest odds ratio, followed by erythropoietin alone. Conclusion: These are promising findings supporting the usability of these biomarkers, especially the systemic immune-inflammation index, as minor criteria in the diagnosis of polycythemia vera. It is especially crucial to note that using erythropoietin in combination with these markers may improve diagnostic accuracy.

## 1. Introduction

Polycythemia is defined as an increase in hemoglobin or hematocrit levels above the reference ranges [1,2]. It has a wide variety of causes, most of which are associated with the development of hyperviscosity, and cases are largely examined as primary or secondary polycythemia (SP). The former is also known as polycythemia vera (PV) and the underlying pathology concerns the bone marrow itself, while the latter is characterized by excessive stimulation of cell production in the normal bone marrow [3].

PV is classified as a clonal myeloproliferative neoplasm (MPN) and is a well-recognized disorder of hematopoietic stem cells [3]. In 2016, the World Health Organization (WHO) revised the diagnostic criteria for PV, which has considerably altered the diagnostic approach [4]. Although these criteria were revised in 2022, no changes were made except that the determination of an increased red cell mass with Cr-labeled red cells has been removed as a diagnostic criterion [5]. PV can cause significant cardiovascular morbidities and mortality [6]. It is extremely important to distinguish PV from SP, as the treatment approaches for these two conditions are very different and particularly because delayed diagnosis might lead to poor outcomes in subjects with PV [7]. Although previous studies support the effectiveness of low erythropoietin (EPO) in differentiating PV from SP [8,9], EPO is a minor indicator with low discriminatory sensitivity [10]. Therefore, easily accessible, low-cost indicators that can reliably and sensitively distinguish PV from SP are required.

Inflammation is one of the most important factors in the development, progression and consequences of MPN, as in many diseases [11,12]. Defective stem cell clones in MPN cause cytokine elevation, thereby perpetuating the inflammatory activity [13]. Some recent studies have shown that cheap and accessible inflammation indices such as the neutrophil-to-lymphocyte ratio (NLR) and platelet-to-lymphocyte (PLR) can distinguish PV from SP, and they have claimed that these markers may be utilized in PV diagnosis [3,7]. However, the evidence supporting these claims is limited. Additionally, the systemic inflammation index (SII), which has recently been shown to be associated with many cancers [14,15,16,17], has not been assessed in PV.

Based on the hypothesis that inflammatory indices might be supportive in the diagnosis of PV, we aimed to investigate the NLR, PLR and SII, as well as their combinations with EPO, in order to assess their roles in distinguishing PV from SP and determine whether they might be superior to EPO alone.

## 2. Materials and Methods

### 2.1. Study Setting and Ethics

This retrospective study was carried out in the Department of Hematology of Bakırköy Dr. Sadi Konuk Training and Research Hospital, İstanbul, Turkey, in line with the principles of the Declaration of Helsinki. Approval was granted by the local ethics committee (decision date: 19 February 2024, decision no: 2024/50). Informed consent was waived because of the retrospective nature of the study.

### 2.2. Study Population and Data Collection

Patients who underwent further examinations due to the detection of polycythemia at our hospital from January 2020 to December 2023 and were then diagnosed with either PV or SP were included in this study. The laboratory thresholds used to diagnose polycythemia were determined according to the 2016 WHO criteria (hemoglobin > 16.5 mg/L for men and >16.0 mg/L for women and/or hematocrit > 49% for men and >48% for women) [4]. Patients with signs of active infection at the time of diagnosis, those with concomitant malignancy and/or autoimmune disease, individuals who had receiving steroids and/or immunosuppressants and/or immunomodulator medication, and subjects with missing data regarding the criteria required for the diagnosis of PV or SP were excluded from the study. All the data for patients, including the polycythemia diagnosis and related features, demographic characteristics, smoking status, laboratory findings and other clinical and laboratory data were collected retrospectively from the hospital database. The diagnosis of PV was made according to the 2016 WHO criteria [4]. Patients with polycythemia who did not meet these criteria were classified as having SP.

#### 2.2.1. Laboratory Analysis

This study incorporated laboratory data derived from blood samples obtained at the time of polycythemia diagnosis. The laboratory parameters included in the investigation comprised a complete blood count, encompassing absolute counts of white blood cells (WBCs), red blood cells (RBCs), lymphocytes, neutrophils, eosinophils, and platelets. Additionally, the hemoglobin, hematocrit, mean corpuscular volume (MCV), erythropoietin, and lactate dehydrogenase (LDH) levels were included. All the analyses were conducted within certified biochemistry laboratories situated within our hospital. The instruments underwent routine calibration, and all the analyses strictly adhered to international standards and the guidelines outlined in the respective kit manuals.

To derive the neutrophil-to-lymphocyte ratio (NLR), the absolute neutrophil count was divided by the absolute lymphocyte count. Similarly, the platelet-to-lymphocyte ratio (PLR) was computed by dividing the absolute platelet count by the absolute lymphocyte count. The systemic immune-inflammation index (SII) was determined by multiplying the platelet count by the neutrophil count, which was then divided by the lymphocyte count [18,19].

#### 2.2.2. Molecular Analyses

DNA was isolated from whole blood with the QIAamp DNA Blood Mini Kit (Qiagen, Hilden, Germany, ID 51104) and the Janus Kinase 2 (JAK2) mutations were assessed by allele-specific polymerase chain reaction, as previously described [20].

#### 2.2.3. Bone Marrow Biopsy and Pathological Analysis

Bone marrow biopsies were conducted, with experienced hematologists employing appropriate procedures. Subsequent to the procedures, all the pathological examinations were undertaken within the pathology laboratories of our hospital under the scrutiny of pathologists proficient in the domain of hematological malignancies.

#### 2.2.4. Smoking Status

Patients who had smoked a minimum of 100 cigarettes throughout their lifetime and were presently consuming at least one cigarette daily were categorized as “active smokers.” Those who had smoked at least 100 cigarettes in their lifetime but had stopped smoking for the past 30 days were categorized as “ex-smokers.” Patients who had smoked fewer than 100 cigarettes in their lifetime and had not smoked within the past 30 days, as well as those who had never smoked, were classified as “non-smokers” [1].

### 2.3. Statistical Analysis

A significance level of 0.05 was employed for all the statistical tests. The analyses were conducted using SPSS software version 25.0 (IBM Corp., Armonk, NY, USA) and MedCalc Statistical Software version 15.8 (MedCalc Software bvba, Ostend, Belgium). The normal distribution of the variables was assessed through histograms and quantile-quantile plots (Q–Q plots). Descriptive statistics were presented as the mean ± standard deviation for normally distributed continuous variables, the median (25th percentile–75th percentile) for non-normally distributed continuous variables, and the frequency (percentage) for categorical variables. Between-group comparisons of the continuous variables were performed using Student’s *t*-test or the Mann–Whitney U test based on the normality assumptions. The categorical variables were analyzed using the chi-square test, Fisher’s exact test, or the Fisher–Freeman–Halton test.

The diagnostic performance of the variables for PV was assessed using a receiver operating characteristic (ROC) curve analysis. The optimal cut-off points for the EPO, NLR, PLR, and SII were determined using the Youden index. Combinations of variables were evaluated using the predicted group membership results obtained from the logistic regression models. Comparisons of the area under ROC curves (AUC) were conducted utilizing the Hanley and McNeil approach. Additionally, logistic regression analyses were performed to calculate the odds ratios to determine the effect sizes regarding their association with PV, both directly and after adjusting for age and sex.

## 3. Results

A total of 229 patients diagnosed with polycythemia were examined, comprising 84 individuals with SP and 145 with PV. The mean age in the SP group was 44.67 ± 15.59, whereas the mean age in the PV group was 56.78 ± 13.30 (*p* < 0.001). Male patients constituted 80.95% of the SP group and 66.21% of the PV group, revealing a significant difference in the gender distribution between the two groups (*p* = 0.026).

The prevalence of splenomegaly was markedly higher in the PV group compared to the SP group, with no cases with splenomegaly in patients with SP (*p* < 0.001). Analysis of the hematological parameters revealed that the PV group exhibited significantly elevated WBC, neutrophil, eosinophil, and platelet counts, as well as RBC, hematocrit, and LDH levels, compared to the SP group. Conversely, the MCV, lymphocyte count, and EPO levels were significantly lower in the PV group compared to the SP group (*p* < 0.001 for all). Finally, we found that the NLR, PLR, and SII were higher in the PV group compared to those with SP (*p* < 0.001 for all).

A total of 227 patients, 82 from the SP group and 145 from the PV group, were examined for the JAK2 V617F mutation. None of the patients in the SP group showed positivity for JAK2 V617F, while 126 patients in the PV group (86.90%) tested positive for JAK2 V617F. Additionally, a total of 67 patients, 43 from the SP group and 24 from the PV group, were examined for the JAK2 exon 12 mutation. None of the patients in the SP group showed positivity for JAK2 exon 12, while four patients in the PV group (16.67%) tested positive for JAK2 exon 12. As expected, post-polycythemia myelofibrosis was not observed in any patient within the SP group, whereas it was detected in three (2.07%) patients within the PV group (*p* < 0.001) (Table 1).

**Figure 1:**
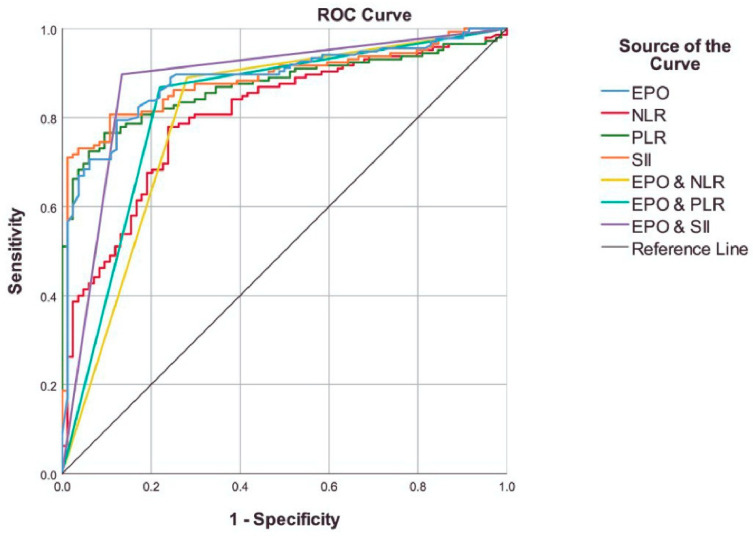
ROC curves of the variables and combinations used to predict polycythemia vera.

We found that an EPO value of <4.85 could significantly predict PV, with 79.41% sensitivity and 87.80% specificity [AUC = 0.886 (0.841–0.931), *p* < 0.001]. The inflammation indices also demonstrated considerable diagnostic accuracy, which is detailed in Table 2. Notably, an SII value of ≥803 demonstrated 80.69% sensitivity and 89.29% specificity [AUC = 0.885 (0.841–0.929), *p* < 0.001]. The combined variables also showed high overall accuracy similar to EPO; however, the Hanley and McNeil analysis showed that the NLR and the EPO and NLR combination had significantly worse classification capabilities compared to EPO alone. It is crucial to note that, despite having a similar AUC value to EPO, the EPO and SII combination yielded improved diagnostic potential, with 88.53% accuracy, 89.71% sensitivity and 86.59% specificity [AUC = 0.881 (0.829–0.933), *p* < 0.001] (Table 2, Figure 1).

Multivariable logistic regression revealed that all the examined parameters, either alone or in combination, exhibited significant performance in predicting PV after adjusting for age and sex (Table 3).

## 4. Discussion

The current retrospective cohort study revealed that inflammation indices calculated from easily accessible laboratory data were capable of discriminating PV from SP. Although the NLR and EPO plus NLR had significantly lower AUC values compared to EPO alone, other parameters and combinations resulted in similar diagnostic potential. Furthermore, the EPO and SII combination had marginally higher overall accuracy in classifying patients into the PV and SP groups, which is a notable advantage. If confirmed by further studies, these results could have implications for the diagnostic use (potentially as a minor criterion) of inflammation indices such as the SII in patients who present with polycythemia.

It is important to distinguish PV from other reasons for erythrocytosis, because early diagnosis and treatment of PV can lead to the prevention of many vascular complications [3]. However, the diagnosis of PV is challenging and often necessitates high-cost and time-consuming laboratory studies, specialized equipment and personnel, and invasive procedures, including bone marrow examinations. In PV, unlike other disorders that cause erythrocytosis, it is well known that the plasma volume increases in parallel with the red cell mass. Therefore, peripheral blood hematocrit and hemoglobin values are unable to reflect the actual red cell volume/burden in the body [21]. EPO is a measure that partially addresses this problem, which results in its use as the only minor criterion for PV diagnosis [4]. The inherent limitations of EPO results explain the relatively low sensitivity and specificity for distinguishing PV from SP (reported as 68% and 94%, respectively) [10]. Patients with PV may have normal EPO levels, potentially leading to misdiagnosis and limiting the diagnostic precision [3,4,12]. In fact, the EPO levels may be normal in approximately one-third of patients with PV. In particular, obese patients, smokers, and those with chronic obstructive pulmonary disease are at high risk of false negative results [22].

It is well known that inflammation triggers all the stages of tumor growth, including initial genetic mutation, tumor development, metastasis, and progression [18]. Similarly, data show that chronic inflammation plays a critical role in the pathogenesis of MPN and that inflammatory conditions may lead to MPN-induced complications [23]. The close relationship between inflammation and MPN pathogenesis offers a potential diagnostic advantage. It may be plausible to utilize inflammatory markers in conjunction with EPO measurements. While PV typically presents with classical features such as erythrocytosis, leukocytosis, and thrombocytosis, it can also manifest as isolated erythrocytosis, isolated thrombocytosis, isolated leukocytosis, or any combination of these. Consequently, inflammation indices derived from inflammation-associated cell counts may offer diagnostic value for PV and could yield several advantages relative to the use of isolated cell counts.

The SII is a relatively new and increasingly popular inflammation marker that is based on peripheral neutrophil, platelet, and lymphocyte counts [24]. The SII has been reported to be a prognostic indicator in various solid organ malignancies, such as hepatocellular carcinoma [25], pancreatic cancer [14], breast cancer [15], lung cancer [16], and gastrointestinal cancer [17]. However, the relationship between the SII and MPN has not been adequately investigated. Ersal et al. evaluated the relationship between myelofibrosis and the SII but did not detect a significant relationship between the SII and mortality [18]. In the current study, we investigated the relationship between the SII and PV diagnosis. The results showed that an SII of ≥803 had higher sensitivity, specificity, and accuracy, positive predictive value (PPV), and negative predictive value (NPV) in detecting PV compared to EPO alone (<4.85), with very similar AUC and OR values. When EPO and the SII were evaluated together (EPO and SII), the diagnostic measures and overall accuracy (88.53%) were improved compared to both EPO (82.57%) and the SII (83.84%) alone, albeit it should be noted that the difference in the AUC value was not significant. Additionally, the variable with the highest OR related to PV detection was EPO and SII. Despite the statistical similarity, these results are very valuable because the role and power of EPO in the diagnosis of PV needs to be improved. The SII may be a parameter that addresses the low sensitivity of EPO. These results, of course, need to be supported by other studies.

Many studies have shown that inflammation indices such as the NLR and PLR have diagnostic and prognostic value for various infectious diseases, inflammatory conditions, surgical emergencies, postoperative complications and various cancers [19,26,27,28]. Some recently published studies have also shown a relationship between these markers and MPNs. For example, Kwon et al. showed that the NLR was higher in patients with essential thrombocytopenia compared to controls [23]. Krečak and colleagues reported that a high PLR at disease diagnosis could identify PV patients at high risk of future thrombosis and death [29]. In another study by the same group, the authors suggested that the NLR should be explored for its role as a prognostic biomarker in essential thrombocytopenia and PV [30]. In a study by Lucijanic et al., the prognostic value of the NLR and PLR in primary myelofibrosis was investigated. The NLR and PLR were found to be higher in patients with primary myelofibrosis than healthy individuals. A higher NLR was associated with JAK2 mutation, wild-type Calreticulin, older age, higher leukocyte count, higher hemoglobin, and larger spleen size. Moreover, a higher NLR and lower PLR were independent markers of poor survival [31]. Zhou et al. showed that the NLR may have a significant prognostic role regarding future thrombosis in essential thrombocythemia patients [32]. The complications that can arise from PV have also been associated with inflammation indices; for instance, the NLR was described as being a prognostic biomarker of venous thrombosis in patients with PV [33]. Similarly, a high NLR was reported to be an independent risk factor for thrombosis progression in PV [34]. On the contrary, there are a number of studies reporting that the NLR does not have a prognostic or diagnostic role in MPNs [35,36]. However, there are few studies examining the roles of the NLR and PLR in distinguishing between PV and SP. We performed this analysis and also compared their diagnostic performances with EPO.

Despite the fact that the NLR (cut-off ≥ 2.35) had the poorest diagnostic performance measures and lower AUC and OR for predicting PV, it still exhibited statistically significant predictive ability in both the ROC and regression analyses. Although combining the NLR with EPO proved more successful than NLR alone, it did not render EPO and NLR the most valuable predictor. Notably, a PLR ≥ 135 displayed the highest specificity and PPV, and when combined with EPO, the sensitivity, accuracy, NPV, AUC, and OR were found to be increased. A recent retrospective study demonstrated significantly elevated NLR and PLR levels in patients with PV compared to SP. Furthermore, the NLR and PLR exhibited a notably higher AUC value than EPO for PV diagnosis, and the combined parameters (NLR and EPO or PLR and EPO) were found to have significantly enhanced diagnostic value compared to EPO alone [3]. In another investigation, researchers examined the diagnostic utility of various parameters in discriminating PV and SP, including the total leukocyte count, neutrophil count, lymphocyte count, platelet count, NLR, and PLR. The findings showed that a PLR cut-off value of >138.1 exhibited the best performance in terms of the AUC, sensitivity, and specificity for diagnosing PV. Furthermore, the study highlighted the necessity of extremely high cut-off values of the NLR for its effective use in PV diagnosis [7]. When the results of existing studies are examined together with those of the current study, it appears that the PLR is a more valuable biomarker than the NLR in the diagnosis of PV, but the evidence is insufficient to recommend the use of the NLR and PLR instead of EPO. We think that the results from the available literature are encouraging for more comprehensive studies and promising for the detection of more sensitive and easily accessible biomarkers in the diagnosis of PV. It is evident, however, that prospectively designed studies are required to confirm these results and that there is also a need for longitudinal records of inflammation indices to understand when they prove to have greater predictive ability.

This study provides valuable information regarding the relationship between the SII and PV and provides meaningful results for the potential utilization of the SII as an alternative or supportive biomarker to EPO. The findings largely support a small number of previous studies that have assessed the NLR and PLR for this purpose, and it appears that the SII demonstrates considerable superiority in this context. Some important limitations of the study should be taken into consideration. The most important of these is the retrospective data collection from a single center. Therefore, the external validity is limited and the disadvantages of a retrospective study are evident, including the fact that data collection was based on measures performed during the routine assessment of patients, not with the precise purpose of addressing the current hypothesis. Secondly, genetic analysis results and/or EPO levels were not obtained from some of the patients included in the study because they were not required. This has led to differences in the number of patients for whom data on the compared variables are available. Although patients with known active infection at the time of blood collection were excluded, it is not possible to be certain in a retrospective study. This may have affected the levels of inflammatory markers, which could also change based on other factors. Another important limitation is that we did not record the body mass index or detailed comorbidity data and the medication records could have been limited during the initial data collection. Lastly, the application of some exclusion criteria, including malignancy (which can trigger erythropoietin release) and drug use (especially steroids can stimulate erythrocytosis), may have led to a reduction in the number of patients with SP, but if this had not been performed, it would have created other limitations for the study, such as ignoring some factors that affect the levels of inflammation markers.

## 5. Conclusions

In summary, the NLR, PLR, and SII demonstrated significant capability in distinguishing PV from SP. When combined with EPO, the diagnostic efficacy of these three variables in identifying PV improved. EPO and SII, followed by the SII alone, emerged as the most valuable biomarker for PV diagnosis. These findings hold promise for the use of these biomarkers, particularly the SII, as supportive parameters or minor criteria in the diagnosis of PV. We believe there is a need for further studies that examine the diagnostic improvement obtained by employing the SII (and other inflammation indices) together with EPO measurements in patients with polycythemia, which could prove crucial for early or easier diagnosis of PV.

## Figures and Tables

**Table 1:** Summary of variables with regard to diagnosis.

	Diagnosis		
	Secondary Polycythemia (n = 84)	Polycythemia Vera (n = 145)	*p*
Age (n = 229)	44.67 ± 15.59	56.78 ± 13.30	<0.001 ^a^
Sex (n = 229)			
Male	68 (80.95%)	96 (66.21%)	0.026 ^c^
Female	16 (19.05%)	49 (33.79%)	
Splenomegaly (n = 227)	0 (0.00%)	39 (27.08%)	<0.001 ^c^
Smoking status (n = 201)			
Non-smoker	34 (41.98%)	70 (58.33%)	0.060 ^c^
Ex-smoker	12 (14.81%)	10 (8.33%)	
Smoker	35 (43.21%)	40 (33.33%)	
WBC (×10^3^) (n = 229)	8.11 (6.72–9.93)	10.80 (8.47–12.56)	<0.001 ^b^
RBC (×10^6^) (n = 229)	5.90 ± 0.52	6.60 ± 1.05	<0.001 ^a^
Hemoglobin (g/dL) (n = 229)	18.02 ± 1.03	17.95 ± 1.73	0.696 ^a^
Hematocrit (%) (n = 229)	52.72 ± 3.77	55.31 ± 5.92	<0.001 ^a^
MCV (fL) (n = 229)	89.19 ± 5.32	85.12 ± 9.20	<0.001 ^a^
Lymphocyte (×10^3^) (n = 229)	2.43 (2.12–2.90)	2.06 (1.65–2.66)	<0.001 ^b^
Neutrophil (×10^3^) (n = 229)	4.58 (3.70–6.49)	7.27 (5.38–8.95)	<0.001 ^b^
Eosinophil (×10^3^) (n = 229)	0.16 (0.09–0.26)	0.27 (0.18–0.42)	<0.001 ^b^
Platelet (×10^3^) (n = 229)	228.5 (195.5–273.5)	407 (301–615)	<0.001 ^b^
LDH (mg/dL) (n = 224)	197 (166–221)	260 (209–345)	<0.001 ^b^
Erythropoietin (mU/mL) (n = 218)	8.00 (6.20–11.50)	2.10 (1.20–4.25)	<0.001 ^b^
NLR (n = 229)	1.92 (1.51–2.35)	3.29 (2.40–4.88)	<0.001 ^b^
PLR (n = 229)	94.37 (78.72–114.06)	216.85 (136.65–290.42)	<0.001 ^b^
SII (×10^3^) (n = 229)	432.33 (335.97–582.93)	1479.11 (872.41–2526.75)	<0.001 ^b^
* JAK2 V617F positivity (n = 227)	0 (0.00%)	126 (86.90%)	<0.001 ^c^
* JAK2 exon 12 positivity (n = 67)	0 (0.00%)	4 (16.67%)	0.014 ^d^
Thrombosis history (n = 229)	13 (15.48%)	37 (25.52%)	0.108 ^c^
Bone marrow biopsy (n = 229)			
No CMPD findings	84 (100.00%)	0 (0.00%)	<0.001 ^e^
PV findings	0 (0.00%)	142 (97.93%)	
Post-polycythemia MF	0 (0.00%)	3 (2.07%)	

Descriptive statistics are presented by using the mean ± standard deviation for normally distributed continuous variables, the median (25th percentile–75th percentile) for non-normally distributed continuous variables and the frequency (percentage) for categorical variables. (a) Student *t* test, (b) Mann–Whitney U test, (c) chi-square test, (d) Fisher’s exact test, (e) Fisher–Freeman–Halton test. * JAK2 mutations were assessed by allele-specific polymerase chain reaction. Abbreviations; CMPD: Chronic myeloproliferative diseases, LDH: Lactate dehydrogenase, MCV: Mean corpuscular volume, NLR: Neutrophil-to-lymphocyte ratio, PLR: Platelet-to-lymphocyte ratio, RBC: Red blood cell, SII: Systemic immune-inflammation index, WBC: White blood cell.

**Table 2:** Performance of the variables and combinations to predict polycythemia vera, ROC curve analysis results.

	Cut-off	Sensitivity	Specificity	Accuracy	PPV	NPV	AUC (95% CI)	*p* ^a^	*p* ^b^
EPO	<4.85	79.41%	87.80%	82.57%	91.53%	72.00%	0.886 (0.841–0.931)	<0.001	-
NLR	≥2.35	77.93%	76.19%	77.29%	84.96%	66.67%	0.803 (0.745–0.861)	<0.001	0.018
PLR	≥135	76.55%	90.48%	81.66%	93.28%	69.09%	0.871 (0.825–0.917)	<0.001	0.709
SII	≥803	80.69%	89.29%	83.84%	92.86%	72.82%	0.885 (0.841–0.929)	<0.001	0.934
EPO & NLR ^†^	-	88.97%	71.95%	82.57%	84.03%	79.73%	0.805 (0.739–0.870)	<0.001	0.010
EPO & PLR ^†^	-	86.76%	78.05%	83.49%	86.76%	78.05%	0.824 (0.762–0.886)	<0.001	0.055
EPO & SII ^†^	-	89.71%	86.59%	88.53%	91.73%	83.53%	0.881 (0.829–0.933)	<0.001	0.883

(a) *p* values for AUC, (b) Comparison of AUC with EPO by using the Hanley & McNeil approach. † Combined variables were evaluated by using predicted group membership of the logistic regression model. Abbreviations; AUC: Area under ROC curve, CI: Confidence intervals, EPO: Erythropoietin, NLR: Neutrophil to lymphocyte ratio, NPV: Negative predictive value, PLR: Platelet to lymphocyte ratio, PPV: Positive predictive value, ROC: Receiver operating characteristic, SII: Systemic immune-inflammation index.

**Table 3:** Odds ratios for polycythemia vera, logistic regression analysis results.

	Unadjusted		Adjusted ^†^	
	OR (95% CI)	*p*	OR (95% CI)	*p*
EPO, <4.85	27.771 (12.715–60.655)	<0.001	29.636 (12.477–70.394)	<0.001
NLR, ≥2.35	11.300 (5.975–21.372)	<0.001	8.768 (4.512–17.038)	<0.001
PLR, ≥135	31.015 (13.611–70.673)	<0.001	27.572 (11.587–65.607)	<0.001
SII, ≥803	34.821 (15.568–77.887)	<0.001	28.109 (12.345–64.006)	<0.001
EPO and NLR	20.693 (10.061–42.558)	<0.001	19.130 (8.860–41.306)	<0.001
EPO and PLR	23.309 (11.338–47.919)	<0.001	28.709 (12.493–65.973)	<0.001
EPO and SII	56.247 (24.230–130.568)	<0.001	48.519 (20.287–116.039)	<0.001

† Adjusted by age and sex. Abbreviations; CI: Confidence interval, EPO: Erythropoietin, NLR: Neutrophil-to-lymphocyte ratio, OR: Odds ratio, PLR: Platelet-to-lymphocyte ratio, SII: Systemic immune-inflammation index.

## Data Availability

All the data are available upon request from the corresponding author.
